# The Impact of Operator Access on Implant Surface Roughness Following Implantoplasty Procedures: A Laboratory Study

**DOI:** 10.1002/cre2.70336

**Published:** 2026-03-18

**Authors:** Eamonn Donohoe, Andreas Krause, Bahman Honari, Lewis Winning, Andreas Stavropoulos, Ioannis Polyzois

**Affiliations:** ^1^ Department of Restorative Dentistry and Periodontology, Dublin Dental University Hospital, Trinity College University of Dublin Dublin Ireland; ^2^ Department of Materials Science and Applied Mathematics Malmö University Malmö Sweden; ^3^ Department of Biostatistics, Dublin Dental University Hospital, Trinity College University of Dublin Dublin Ireland; ^4^ Department of Periodontology, Faculty of Odontology Malmö University Malmö Sweden; ^5^ Department of Periodontology Blekinge Hospital Karlskrona Sweden; ^6^ Division of Conservative Dentistry and Periodontology, University Clinic of Dentistry Medical University of Vienna Vienna Austria; ^7^ Department of Periodontology, School of Dental Medicine University of Bern Bern Switzerland

## Abstract

**Objectives:**

To evaluate the influence of operator access on surface roughness parameters and material loss following implantoplasty (IP).

**Material and Methods:**

A total of 42 dental implants were utilized in this study, and three different implant designs. Two set‐ups were used: a table‐top, allowing unrestricted access, and a phantom‐head, imposing limitations in access and better replicating the clinical situation. A combination of tungsten carbide burs and silicone polishers was used to perform IP on the implants. The total amount of time required for IP of each implant was measured for comparisons between modalities, and a 2D roughness profile analysis (*R_a_* and *R_z_*) was conducted using a contact stylus profilometer. The weight and the diameter of the implants were also measured before and after IP was performed.

**Results:**

A statistically significant difference was found between the *R_a_* (*p* = 0.004) and *R_z_* (*p* = 0.019) values of implants prepared under table‐top conditions, being smoother, when compared to the ones prepared in the phantom head. The time required to perform IP was significantly different between the two different settings (*p* < 0.001) and between some implant types. The set‐up in which IP was performed did not have a significant impact on the change in implant mass (*p* = 0.926) or in implant diameter (*p* = 0.721).

**Conclusions:**

Implants treated with IP in the phantom‐head set‐up exhibited significantly greater surface roughness and required longer procedural time compared to those treated in the table‐top model, but did not influence material loss.

## 1 Introduction

Peri‐implantitis is a biofilm‐associated pathological condition affecting dental implants, characterized by inflammation of the surrounding connective tissue and progressive bone loss (Al Ghazal et al. 2017; Berglundh et al. 2018; Schwarz et al. 2018). Lee et al. (2017) found a mean peri‐implantitis prevalence of 9.25% at the implant level, and 19.83% at the subject level, and epidemiological projections suggest a continued rise in implant‐related diseases (Madianos et al. 2016).

The “Prevention and treatment of peri‐implant diseases—The EFP S3 level clinical practice guideline” recommends an initial non‐surgical step of therapy, including oral hygiene instructions, risk factor control, prosthesis maintenance, and modification as required, along with supra‐ and sub‐marginal instrumentation (Herrera et al. 2023). If the endpoints of non‐surgical therapy are not achieved, surgical therapy involving access flaps, resective surgery, or regenerative surgery is recommended (Donos et al. 2023; Herrera et al. 2023; Karlsson et al. 2023).

Implantoplasty (IP) is an adjunctive approach for the surgical management of peri‐implantitis; it is considered a form of resective therapy aiming to smoothen the exposed implant surface (Schwarz et al. 2022), thereby reducing bacterial adhesion and potentially enhancing the effectiveness of patients’ self‐performed oral hygiene (Bertl et al. 2024). Although studies overall indicate successful clinical outcomes, without remarkable complications after IP (Stavropoulos et al. 2019), the current EFP S3 level clinical practice guidelines state there is insufficient evidence to make any recommendation on it's use for the surgical management of peri‐implantitis (Herrera et al. 2023). In this context, the majority of existing in‐vitro studies involving IP, employed a table‐top set‐up on discs, which provided the operators with unobstructed access to the surface to be prepared (grinded down); this set‐up is therefore not representative of the clinical scenario (Tawse‐Smith et al. 2016; Toma et al. 2018; Beheshti Maal and Verket 2022; Yildiz et al. 2022; Vierling et al. 2024). Some studies have indeed evaluated the final surface roughness of implants after IP in a phantom head model (Rimondini et al. 2000; Sahrmann et al. 2019; Burgueño‐Barris et al. 2023). The phantom head model allows many aspects of a clinical setting to be transferred and may provide a better representation of the surface topography likely to be achieved clinically when IP is performed intra‐orally. However, no studies have directly assessed whether surface roughness differs between table‐top and phantom head model's set‐up.

This laboratory study aimed to evaluate the influence of operator access on two surface roughness parameters (*R*_*a*_ and *R*_*z*_) and material loss on dental implant surfaces after IP. *R_a_* (Arithmetic Average Roughness), quantifies the average of the absolute heights of surface irregularities, both peaks and valleys, relative to the mean line across a defined evaluation length, producing a single value that describes overall surface finish. Smaller *R_a_* values correspond to smoother surfaces. *R_z_* (Mean Roughness Depth), on the other hand, characterizes surface texture by averaging the height difference between the five tallest peaks and the five deepest valleys over multiple sampling lengths, reflecting the overall vertical range of surface irregularities. Together, they provide a full picture of both average texture and surface extremes.

Two set‐ups were used: a table‐top, allowing unrestricted access and representing an idealized setting, and a phantom‐head, imposing limitations in access and better replicating the clinical situation. The study also examined whether implant type characteristics, surface microstructure, and implant macro design may affect IP. Finally, for implants prepared in the phantom head model, the buccal and lingual aspects were compared to evaluate access‐related differences.

## 2 Materials and Methods

Given the novelty of the present study, no prior data were available for direct sample size estimation. Therefore, a power calculation was conducted based on the findings of Sahrmann et al. (2019), who evaluated surface roughness (*R_a_* and *R_z_*) between two bur protocols for interproximal (IP) reduction in implant models. For *R_a_*, assuming a standard deviation (SD) of 0.145 μm and an expected mean difference of 0.38 μm, a sample of four experimental and four control specimens would achieve 80% power at a 5% significance level. For *R_z_*, assuming a SD of 0.71 μm and a mean difference of 2.25 μm, three experimental and three control specimens would provide the same power and significance level.

The procedures were conducted in conformity with the 2014 CRIS guidelines for reporting in‐vitro studies (Krithikadatta et al. 2014). A total of 42 dental implants were utilized and analyzed in this study. Three different implant designs were used:

1. 14 Straumann Tissue Level Tapered Effect Implant (ST) SLA 4.1 × 12 mm RN (Institute Straumann AG, Basel, Switzerland)
2. 14 Ankylos Bone Level Implants (ANK) 3.5 × 14 mm (Dentsply Sirona, Hanau, Germany)
3. 14 Sweden and Martina PRAMA FR ZIRTI (S&M) 3.8 × 13 mm (Sweden and Martina SpA, Padua, Italy).

The weight of the implants (in mg) was measured before and after IP was performed using a micro‐weighing precision scale Brifit Digital miniscale (Shenzhen Amier Technology, Guangdoing, China). Additionally, the diameter of each implant was recorded 2 mm apically to the most coronal roughened aspect of the implant before IP and again at the same level after IP, using an electronic calliper (Digital ABS AOS Calliper, Mitutoyo, Sakado, Japan).

A mark was made with a DEHP Bur Diamond FG 109‐010 M bur (Frank Dental GmbH, Gmund am Tegernsee, Germany) on the internal wall of the implants. In the table‐top (control) group, this mark was placed in an arbitrary location, while in the phantom head (test) group, it was made on the buccal aspect to identify it for analysis purposes. The total amount of time required for IP of each implant was also measured using a Garmin smartwatch, Venu 2 Plus (Garmin International Inc., Olathe, USA).

**Figure 1:**
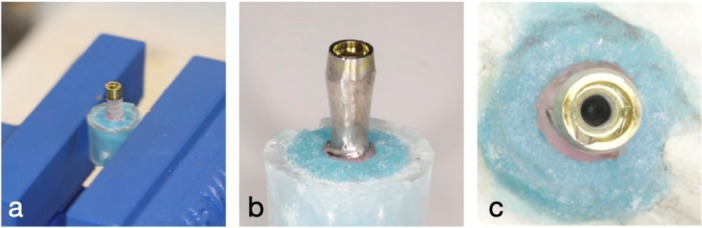
a–c: Photographs showing (a) the placement of an implant pre‐IP in a table‐top vice grip, (b) S&M implant after IP, and (c) presence of a notch on the internal surface of the tabletop implant.

The control group of the dental implants were housed in a polystyrene coping SIGMA‐ALDRICH Scienceware C3858‐50EA (St. Louis, MO63178, USA). This coping was adjusted with the incorporation of a notch to provide mechanical retention of Triad material (Dentsply, Charlotte, North Carolina, USA). Triad acrylic was placed in the coping, and Dentsply conical reamers (Dentsply, Charlotte, North Carolina, USA) were used to prepare the unset Triad. The Triad material within the coping was cured as per manufacturer recommendations, and the implants were then checked with a periodontal probe (1 mm grading) to confirm that 5 mm of the rough surface would remain exposed. The implants were then placed in Impregum Penta Polyether impression material (3 M, Maplewood, Minnesota) and allowed to set as per manufacturer recommendations. Finally, the implants were mounted on the tabletop with a vice (Figure 1a–c).

### 2.1 Preparation of the Casts

**Figure 2:**
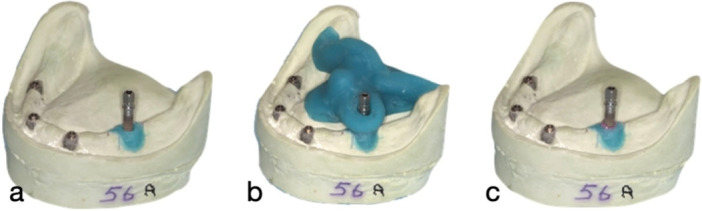
a–c: Photographs showing placement of an ANK implant in the 36 site using the customized Triad (Dentsply, Charlotte, North Carolina, USA) jig on cast model. (a) Implant in situ in cured Triad on the cast, (b) confirmation of location using customized jig, and (c) placement of Impregum Penta Polyether impression material (3 M, Maplewood, Minnesota).

In the test group using phantom heads, implants were positioned in position 36 in identical dental stone casts. The casts had a magnetic base, which allowed them to be (re)mounted in phantom heads. A space was prepared within the cast to house the implant, and a customized positioning jig made from Triad (Dentsply, Charlotte, North Carolina, USA) was used to ensure that all implants were placed in the exact same position in all models (Figure 2a–c).

**Figure 3:**
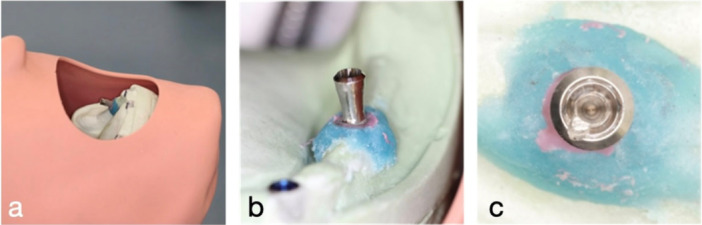
a–c: Photographs showing (a) placement of the cast with an ST implant in the 3–6 position in the phantom head model, (b) the phantom head implant after IP, and (c) presence of a notch on the internal surface of demarcating the buccal surface.

Similarly, to the control group, Dentsply (Dentsply, Charlotte, North Carolina, USA) conical reamers were used to prepare the unset Triad material, which was then cured as per the manufacturer's recommendations. The implants were then checked as above to confirm that 5 mm of the rough surface would remain exposed after the placement of the impression material. Impregum Penta Polyether impression material (3 M, Maplewood, Minnesota) was then placed into the space created, and the implants were inserted into the stone casts (Figure 3a–c).

### 2.2 Implantoplasty

One researcher (E.D.) completed the IP procedures freehand on all implants, until the operator judged that the implants were appropriately smooth by inspection. IP was performed under ×3 magnification using Orascoptic EyeZoom (Madison, Wisconsin USA) loupes with light illumination in the tabletop and phantom head settings. IP of the different implant types and settings was performed in alternating sequences following a flow chart.

The following tungsten carbide burs were used to perform IP on the implants: HM379 (023) and HM379U (023) (Hager and Meisinger GmbH, Neuss, Germany) on a high‐speed handpiece (W&H Dentalwerk, Bürmoos, Austria) with irrigation. The implants were then subsequently polished with a red rubber polisher 9503CA (Sweden and Martina SpA, Padua, Italy) followed by a green rubber polisher 9533CA (Sweden and Martina SpA, Padua, Italy) at 1500 rpm on a slow handpiece (W&H Dentalwerk, Bürmoos, Austria).

After IP, the implants were removed with Crown & Bridge Remover GC KY‐Pliers (Lucerne, Switzerland). The remaining impression material on the implant surface was removed by hand and high‐pressure air.

### 2.3 Experimental Measurements

A 2D roughness profile analysis (*R_a_* and *R_z_*) was conducted using a Surftest Sj‐210 contact stylus profilometer (Mitutoyo Europe GmbH, Neuss, Germany) with a 5 μm diameter diamond and measuring force of 4 mN with a traversing speed of 0.25 mm/s.

**Figure 4:**
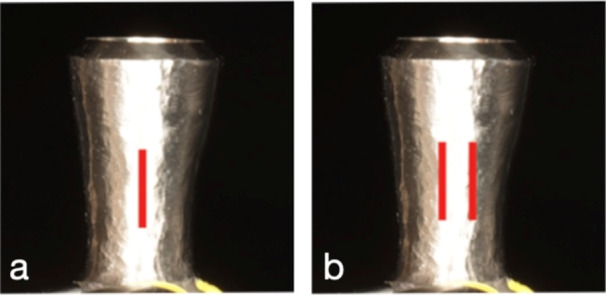
a, b: Diagrammatic representation of surface measurement in (a) table‐top set‐up and (b) phantom‐head set‐up.

For the tabletop implants, two opposing sites were randomly selected on each implant. At each site, four repeated measurements were obtained, and the mean value was calculated. These measurements from these control implants served as comparators with the test group (Figure 4a).

In the phantom head model, two sites on the buccal surface of the implants, indicated by the notch on the internal connection, and 2 mm apart were measured. In addition, two sites on the lingual surface of each implant were also measured. At each site, four repeated surface roughness measurements were taken, and the mean value for each surface (buccal and lingual) was calculated for analysis (Figure 4b).

### 2.4 Scanning Electron Microscopy (SEM)

Eight implants were selected for SEM analysis, after recording the various surface parameters, based on their outlier values—either exceptionally high or low—in *R_a_* or *R_z_* surface roughness measurements, to validate the profilemeter's identification. Before the use of SEM, the implants were cleaned with ethanol, rinsed with deionized water, and left to dry in a clean environment. The eight implants selected were individually mounted on aluminum pin stabs (12.5 mm dia, 3.2 × 8 mm) (Agar Scientific, Rotherham, UK) using conductive carbon adhesive tabs. Conducting Carbon Cement (Leit‐C) (Plano GmbH, Wetzlar, Hessen, Germany) was also used around the tip of the implants to secure the specimens better. A Carl Zeiss EVO LS10 (Zeiss, Oberkochen, Germany) was used to obtain images of the samples at two different magnifications (×50 and ×100).

### 2.5 Statistical Analysis

Data were recorded in a data collection sheet in Microsoft Excel (Redmond, Washington, USA) software. These data were imported to IBM SPSS Statistics for Windows, Version 29.0 (IBM Corp., Armonk, NY, USA). All statistical analyses were performed blindly regarding treatment group using SPSS with significance set at *p* < 0.05.

Descriptive statistics were used to express surface roughness (*R_a_* and *R_z_*) before and after IP, operative time, implant width, and implant mass between the tabletop (control) and phantom head (test) models. A Shapiro–Wilk test was used to check for normality of data. Median values were compared for each of the parameters using a Mann–Whitney *U* Test to determine statistical significance.

A two‐way ANOVA was used to determine the interaction between implant type and setting on key outcome variables, including differences in surface roughness, procedural time, and material loss (width and mass loss). Post‐hoc analysis was conducted with Tukey HSD for pairwise comparisons of implant types. Two‐sided independent samples *t*‐tests were performed to compare the means of the *R_a_*, *R_z_*, width difference and mass difference between the models for each individual implant type, with significant differences reported.

Paired comparisons of surface roughness (*R_a_* and *R_z_*) between buccal and lingual aspects were performed for each implant type using paired samples *t*‐tests or Wilcoxon Signed Rank Tests. In addition, Cohen's *d* was used on the parametric data to determine the effect size.

## 3 Results

A total of 42 implants were used in the study, with 21 implants in the tabletop and 21 implants in the phantom head setting.

The mean (SD) values of surface roughness (*R_a_*/*R_z_*), procedural time, implant mass (pre‐ and post‐operatively), and implant width (pre‐ and post‐operatively) for all implants included in the study are presented in Table 1.

**Table 1:** Display of median and interquartile ranges (IQR) for *R_a_*, *R_z_*, time, mass before and after IP, along with the width before and after IP of all implants in the table‐top and phantom‐head set‐up.

	Table‐top median (IQR)	Phantom‐head median (IQR)	
*R_a_* (μm)	0.44 (0.37–0.62)	0.53 (0.49–0.58)	*p* = 0.004
*R_z_* (μm)	2.47 (1.98–2.75)	2.78 (2.56–3.01)	*p* = 0.019
Time (s)	685 (648–738)	765 (733–800.50)	*p* < 0.001
Mass before (g)	0.43 (0.37–0.62)	0.44 (0.37–0.62)	*p* = 0.623
Mass after (g)	0.40 (0.34–0.56)	0.40 (0.57–0.34)	*p* = 0.725
Width before (mm)	3.81 (3.50–4.37)	3.81 (3.51–4.39)	*p* = 0.367
Width after (mm)	3.00 (2.97–3.26)	3.12 (2.92–2.35)	*p* = 0.821

A statistically significant difference was found between the *R_a_* (*p* = 0.004) and *R_z_* (*p* = 0.019) values of all implants prepared under table‐top conditions, being smoother, when compared to the ones prepared in the phantom head, simulating the clinical environment.

A statistically significant difference was also found between the time (*p* < 0.001) required for implants prepared under table‐top conditions, compared to the ones prepared in the phantom head, with the former ones requiring less time to achieve the same smoothness judged by inspection.

Finally, no statistically significant differences were found in implant mass and diameter between the implants prepared in a table‐top set‐up compared to those in a phantom head, either before or after IP.

### 3.1 Comparison Among Implant Systems (Surface Roughness)

The mean and SD values are presented in Tables 2, 3, 4 for the differing implant types and settings.

**Table 2:** Display of mean and standard deviation (SD) values, for ST implants for *R_a_*, *R_z_*, time, width difference, and mass difference for implants prepared in table‐top and phantom‐head set‐up.

Straumann (*n* = 14)	Tabletop mean, SD	Phantom head mean, SD
*R_a_* (μm)	0.45 (0.09)	0.56 (0.06)
*R_z_* (μm)	2.60 (0.40)	2.94 (0.45)
Time (s)	713.29 (69.58)	791.43 (53.47)
Width difference (mm)	1.07 (0.13)	1.01 (0.08)
Mass difference (g)	0.05 (0.01)	0.05 (0.01)

**Table 3:** Display **of** mean **and** st**andard deviati**on (SD) values, for ANK implants for *R_a_*, *R_z_*, time, width difference, and mass difference for implants prepared in table‐top and phantom‐head set‐up.

Ankylos (*n* = 14)	Tabletop mean, SD	Phantom head mean, SD
*R_a_* (μm)	0.42 (0.12)	0.57 (0.17)
*R_z_* (μm)	2.10 (0.54)	2.94 (0.94)
Time (s)	701.29 (50.10)	788.43 (45.96)
Width difference (mm)	0.45 (0.08)	0.51 (0.14)
Mass difference (g)	0.03 (0.01)	0.04 (0.01)

**Table 4:** Display of mean and standard deviation (SD) values, for S&M implants for *R_a_*, *R_z_*, time, width difference, and mass difference for implants prepared in table‐top and phantom‐head set‐up.

Sweden and Martina (*n* = 14)	Tabletop mean, SD	Phantom head mean, SD
*R_a_* (μm)	0.43 (0.06)	0.49 (0.11)
*R_z_* (μm)	2.41 (0.41)	2.55 (0.57)
Time (s)	671.86 (42.13)	725.43 (60.32)
Width difference (mm)	0.85 (0.03)	0.81 (0.12)
Mass difference (g)	0.037 (0.01)	0.036 (0.01)

Average surface roughness *R_a_* was measured after completing IP, and the *R_a_* values of different implant types and the set‐ups in which IP was performed were compared. An ANOVA test did not find a significant difference among the three implant types (*p* = 0.620). However, the average surface roughness *R_a_* between the table‐top and the phantom model set‐up did reach statistical significance (*p* = 0.006). The interaction effect was not statistically significant (*p* = 0.467). A two‐sided independent sample *t*‐test revealed that a significant difference for *R_a_* of −0.084 μm (95% CI: −0.168 to −0.001) was present for the Straumann implant between the tablet‐top and phantom head model (*p* = 0.048).

Surface roughness *R_z_* was measured after IP, and the *R_z_* of the different implant types and of the set‐up in which the IP was performed were compared. An ANOVA test did not find a significant difference in *R_z_* values among the three implant types (*p* = 0.473). However, the average surface roughness *R_z_* between the table‐top and the phantom model set‐up did reach statistical significance (*p* = 0.023). The interaction effect was not statistically significant (*p* = 0.216). A two‐sided independent sample *t*‐test did not reveal a difference of *R_z_* between any of the implant systems in the phantom head and tabletop models.

### 3.2 Time

The time required to perform IP was recorded during the procedure. An ANOVA test revealed a statistically significant difference in operative time required between the two different settings (*p* < 0.001), along with a statistically significant difference between implant type (*p* = 0.027).

**Figure 5:**
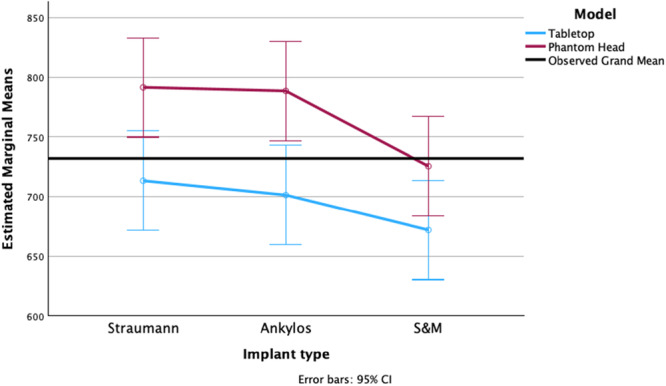
Graphical representation of the time required for IP of the different implant types prepared in a tabletop and phantom head model.

A Tukey HSD post hoc test found significant differences in time required among the implant types, with ST implants requiring significantly more time, 53.71 s, for IP compared to S&M implants (95% CI; 3.49, 103.94; *p* = 0.034). Additionally, S&M implants took 46.21 s less time to prepare compared to ANK implants (95% CI; −4.01, 96.44; *p* = 0.077). There was no statistically significant difference in operative time required between ST implants compared to ANK implants, with a difference of 7.50 s (95% CI; −57.73, 42.73; *p* = 0.929). The interaction effect was not statistically significant (*p* = 0.702) (Figure 5).

A two‐sided independent sample *t*‐test revealed a significant difference for time taken between ST implants on the table‐top and the phantom head set‐up of −78.14 s (95% CI; −150.41 to −5.877; *p* = 0.036). In addition, a significant difference of −87.14 s was also found for ANK implants (95% CI; −143.13 to −31.15; *p* = 0.005).

### 3.3 Diameter and Weight

Following the IP procedures, the diameter of the implants was measured at the previously specified sites, and these values were compared to the measurements taken before IP. An ANOVA test revealed a statistically significant difference in the reduction of implant diameter among the three implant types (*p* < 0.001). However, the set‐up in which the IP was performed did not have a significant impact on the change in implant diameter (*p* = 0.721).

**Figure 6:**
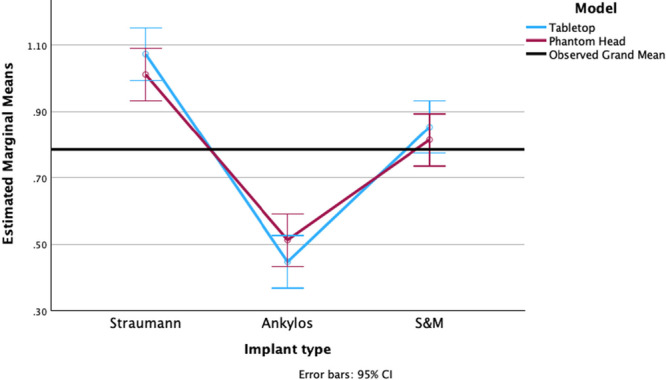
Graphical representation of the difference in diameter after IP of the different implant types prepared in a table‐top and phantom‐head set‐up.

A Tukey HSD post hoc analysis revealed that ST implants exhibited the greatest mean diameter change following IP, significantly exceeding both ANK (0.5621 mm, 95% CI [0.4670, 0.6572], *p* < 0.001) and S&M implants (0.2086 mm, 95% CI [0.1135, 0.3037], *p* < 0.001). ANK implants demonstrated the smallest reduction in diameter, which was significantly lower than S&M (−0.3536 mm, 95% CI [−0.4487, −0.2585], *p* < 0.001). The interaction effect was not statistically significant (*p* = 0.233). Two‐sided independent sample *t*‐tests comparing the individual implant types in the two settings for diameter difference were not statistically significant (*p* > 0.05) (Figure 6).

After IP was performed on the implant, the mass of the implants was measured, and these values were compared to the measurements taken before IP. An ANOVA test revealed a statistically significant difference in the reduction of implant width among the three implant types (*p* < 0.001). However, the set‐up in which the IP was performed did not have a significant impact on the change in implant mass (*p* = 0.926).

**Figure 7:**
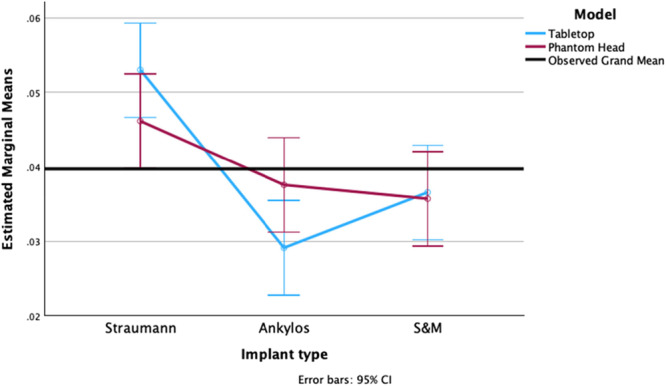
Graphical representation of the difference in weight after IP of the different implant types prepared in a table‐top and phantom‐head set‐up.

A Tukey HSD post hoc test found ST implants had a significantly greater mass reduction compared to both ANK (0.0162 g, 95% CI [0.0086, 0.0239], *p* < 0.001) and S&M (0.0134 g, 95% CI [0.0058, 0.0211], *p* < 0.001). No significant difference in mass reduction was found between ANK and S&M dental implants (−0.0028 g, 95% CI [−0.0104, 0.0049], *p* = 0.649). The interaction effect was not statistically significant (*p* = 0.06). Two‐sided independent sample *t*‐tests comparing the individual implant types in the two set‐ups for mass loss were not statistically significant (*p* > 0.05) (Figure 7).

Finally, no significant differences in *R_a_* or *R_z_* were identified between the buccal and lingual surfaces overall for neither of the implant systems.

### 3.4 Scanning Electron Microscopy

**Figure 8:**
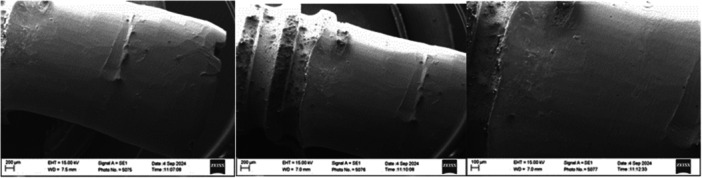
SEM images of Implant 5. Coronal area of IP under ×50 magnification, apical area of IP under ×50 time magnification, and apical area of IP under ×100 magnification.

Implant no. 5 (ANK, Lingual surface) showed the highest surface roughness in the phantom head group and throughout the study (*R_a_* = 1.09 µm, *R_z_* = 5.98 µm). SEM images displayed a coarse and irregular surface, with a prominent ridge in the center of the treatment implant surface. These features are consistent with the elevated roughness measurements observed (Figure 8).

**Figure 9:**
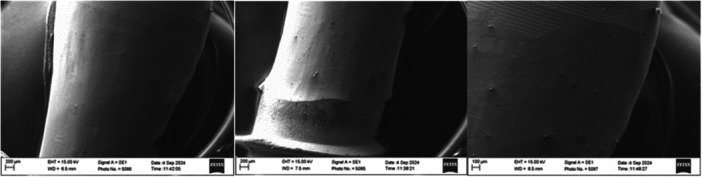
SEM images of Implant 42. Coronal area of IP under ×50 magnification, apical area of IP under ×50 magnification, and coronal area of IP under ×100 magnification.

Implant no. 42 (S&M, Buccal surface, Phantom Head) had low surface roughness values (*R_a_* = 0.38 µm, *R_z_* = 1.81 µm). SEM images show a smoothened implant surface with fine lines, isolated surface pitting, and protrusions (Figure 9).

## 4 Discussion

The results of the study herein show a significantly increased surface roughness on implants prepared in the phantom head set‐up compared to those in the table‐top set‐up, indicating an impact of operator access on the outcome of IP regarding surface smoothness. These findings agree with prior studies demonstrating that IP significantly reduces implant surface roughness relative to the original roughness (Ramel et al. 2016; Camps‐Font et al. 2023; Tsampli et al. 2024). The surface roughness values achieved herein were markedly lower than the *R_a_* and *R_z_* found by Rimondini et al. (2000) and Sahrmann et al. (2019), with both of these studies using a phantom head design. A study by Burgueño‐Barris et al. (2023) who also used a phantom head could not be directly compared to our findings, as they used other surface outcome measures (i.e., Sa and Sz), which are not directly comparable to those used herein (Dagnall 2011).

A similar bur protocol of tungsten carbide burs and silicone polishers was used to perform IP on discs in an in vitro study by Yildiz et al. (2022) in which they reported surface roughness values ranging between 0.13 and 0.14 µm (*R_a_*) and a *R_z_* of 0.69 µm. While our study demonstrated relatively low roughness values within a phantom head set‐up, as mentioned above, the smoother surfaces observed in disc‐based tabletop models using a similar bur sequences are noteworthy. This discrepancy may be attributed to enhanced instrumentation access or the inherently flat geometry of discs, which contrasts with the complex macrostructure of dental implants that may hinder uniform surface finishing. Indeed, studies which used IP as a treatment modality in a table‐top set‐up on discs provide the operator with unobstructed access to the surface allocated for treatment (Tawse‐Smith et al. 2016; Toma et al. 2018; Beheshti Maal and Verket 2022; Yildiz et al. 2022; Vierling et al. 2024). All IP procedures result in a substantial reduction of surface roughness compared to sandblasted, acid‐etched surfaces or other surfaces of similar roughness, which suggests that baseline surface characteristics have a limited impact on the final surface roughness following IP. (Tawse‐Smith et al. 2016; Toma et al. 2018; Beheshti Maal and Verket 2022; Yildiz et al. 2022; Vierling et al. 2024). IP with a polishing bur sequence typically achieves *R_a_* values below 0.5 μm and Sa values of 0.2–0.4 μm (Tawse‐Smith et al. 2016; Beheshti Maal and Verket 2022; Yildiz et al. 2022). Tungsten carbide burs, followed by silicone polishers, appear to produce the smoothest *R_a_* values (< 0.2 μm) (Yildiz et al. 2022). Without a polishing sequence, the average surface roughness is significantly higher (Tawse‐Smith et al. 2016; Toma et al. 2018; Beheshti Maal and Verket 2022; Yildiz et al. 2022). Vierling et al. (2024) noted that diamond burs tend to leave more irregular, rougher surfaces compared to tungsten carbide burs.

The use of phantom head models allows researchers to emulate clinical conditions, providing an assessment of IP in a simulated environment (Rimondini et al. 2000; Sahrmann et al. 2019; Burgueño‐Barris et al. 2023). Rimondini et al. (2000) used a phantom head model on plasma‐sprayed implants using bladed tungsten carbide burs and diamond burs of various grits. Diamond burs with reducing grit sizes of 30 μm and 15 μm achieved an *R_a_* of 1.04 ± 0.14 μm and *R_z_* of 4.86 ± 0.57 μm, while a combination of carbide burs achieved a comparable *R_a_* of 1.16 ± 0.26 μm and *R_z_* of 4.04 ± 0.83 μm (Rimondini et al. 2000). In this context, surface roughness of the buccal and lingual surfaces of implants treated in the phantom head set‐up, overall, did not show any significant differences for *R_a_* and *R_z_*. These findings are in agreement with the results of the study by Burgueño‐Barris et al. (2023), who found that using a phantom‐head model, the surface roughness achieved on different aspects of an implant was similar.

Overall, clinicians should aim to remove as little material as necessary to achieve a smooth surface after IP, to avoid compromising the mechanical properties of the implant. Our findings suggest that material loss across the set‐ups does not differ, although differences are present when different implants are used. Prior to IP of implants, macro‐geometry and width should be considered to plan the IP and to account for potential complications such as the risk of implant fracture in narrow diameter implants (Chan et al. 2013; Bertl et al. 2021; Stavropoulos et al. 2023). No significant difference in mass reduction after IP was observed between implants prepared in the table‐top and in the phantom head set‐up (0.03–0.04 g). This was unexpected, as greater mass loss was anticipated in the phantom head model due to restriction in access and sub‐optimal visual control during IP; the longer operative time may have however, compensated for this, i.e., the operator was more careful. The mean mass reduction in this study was slightly higher than that reported by Sahrmann et al. (2019); (0.02–0.025 g), possibly due to differences in implant type or diamond bur protocol. Nonetheless, the mass loss observed with tungsten carbide burs aligns with previous findings (Sivolella et al. 2021; Tsampli et al. 2024).

The average time required for IP was significantly increased in the phantom head model (765 s) compared to the table‐top set‐up (685 s) (*p* < 0.001). These results were similar to those of Sahrmann et al. (2019) and Jorio et al. (2021). However, in contrast to Sahrmann et al. (2019), our study did not have an allocated amount of time for the use of the two tungsten carbide burs and polishing with silicone carbide polishers, and so the total time required to perform IP was measured. Of interest is the finding that although the preparation time was longer for the phantom head model implants, this did not lead to a further reduction in *R_a_* or *R_z_*, which in turn implies that with a given bur/polisher combination, increased IP time does not necessarily result in a smoother surface.

The study herein took a preliminary step toward assessing how implant macro‐design may affect IP outcomes by using implant systems with different macro‐geometry, i.e., collar and thread design. The implants differed from one another, being either tissue‐level with a divergent or convergent neck, or bone‐level, as well as regarding their thread pitch, depth, and shape. However, our study failed to show any difference in the final surface roughness achieved for *R_a_* (*p* = 0.620) and *R_z_* (*p* = 0.473) for the implant types assessed. This finding may allude to the possibility that the final surface roughness of an IP procedure is independent of baseline implant surface roughness and treatment, along with the macro geometry of the implant, and instead it is the cutting means (i.e., burs) that is important. However, only three different types of implants were used herein, and generalizing the results to all existing implant systems is not possible.

There are a number of limitations to our study which may have influenced the results. First, the in vitro design, conducted using phantom heads, was chosen to restrict operator access and standardize conditions. However, this approach does not fully replicate the clinical challenges and complexities encountered in real patients. The implants in the study herein did not have implant restorations present, and all the implants were affected by a 5 mm horizontal implant defect with no adjacent teeth. This limits the generalizability of our study to cases which may have those factors present in clinical practice. It has already been shown that the presence of a crown can result in increased residual threads in a phantom head study with a soft tissue model present (Burgueño‐Barris et al. 2023). A single operator performed IP of all implants in our study. Although a sole operator was used to limit inter‐operator variability, this also reduces the generalizability of the results. Individual operator skill and technique vary, with some clinicians potentially being able to achieve improved results. In addition, the application of pressure was not controlled for when performing IP on the dental implants on the phantom head and tabletop models. In this way, it was attempted to reproduce the real‐world conditions present in a patient's mouth. However, this lack of pressure control means that our outcomes, while generally repeatable by the same operator, might differ if another operator performs the procedure.

Future research should investigate how the presence of different morphologies of peri‐implant defects, the presence of implant restoration, and the use of a soft tissue model would affect outcomes of IP.

## 5 Conclusion

Implants treated with IP in the phantom‐head set‐up exhibited significantly greater surface roughness and required a longer time compared to those treated in the table‐top model, but did not influence material loss. The increased duration is likely due to the added complexity and restricted access inherent in a simulated intraoral environment, as opposed to the freer table‐top set‐up.

## Author Contributions

Ioannis Polyzois contributed to the conception and design of the study, contributed to data collection, and critically revised the manuscript. Eamonn Donohoe contributed to the data collection, organized the database, and wrote the first draft of the manuscript. Andreas Krause contributed to data collection. Lewis Winning contributed to the conception and design of the study and critically revised the manuscript. Bahman Honari performed the statistical analysis. Andreas Stavropoulos contributed to the design of the study and critically revised the manuscript.

## Conflicts of Interest

The authors declare no conflicts of interest.

## Data Availability

Data available on reasonable request from the authors.
